# The management pattern and outcomes of chronic thromboembolic pulmonary hypertension: rationale and design for a Chinese real-world study

**DOI:** 10.1186/s12890-024-03042-5

**Published:** 2024-06-03

**Authors:** Shuai Zhang, Yishan Li, Yuanhua Yang, Sugang Gong, Zhenwen Yang, Cheng Hong, Xiaopei Cui, Jun Wan, Yingqun Ji, Hong Chen, Ling Zhu, Chenghong Li, Zhaozhong Cheng, Yunhui Zhang, Qiguang Wang, Qin Luo, Lu Guo, Guofeng Ma, Baoshi Han, Zhihong Liu, Changming Xiong, Lan Wang, Qiushang Ji, Fajiu Li, Shengfeng Wang, Zhu Zhang, Yunxia Zhang, Dingyi Wang, Qian Gao, Qiang Huang, Wanmu Xie, Zhenguo Zhai, Chen Wang

**Affiliations:** 1 National Center for Respiratory Medicine, State Key Laboratory of Respiratory Health and Multimorbidity, National Clinical Research Center for Respiratory Diseases, Institute of Respiratory Medicine, Chinese Academy of Medical Sciences, Department of Pulmonary and Critical Care Medicine, Center of Respiratory Medicine, China-Japan Friendship Hospital, Beijing, P.R. China; 2 Department of Pulmonary and Critical Care Medicine, Beijing Chaoyang Hospital, Capital Medical University, Beijing, P.R. China; 3 Dpartments of Pulmonary Circulation, Shanghai Pulmonary Hospital, Tongji University School of Medicine, Shanghai, P.R. China; 4 Department of Cardiology, Tianjin Medical University General Hospital, Tianjin, P.R. China; 5 State Key Laboratory of Respiratory Diseases, Guangdong Key Laboratory of Vascular Diseases, Guangzhou Institute of Respiratory Health, The First Affiliated Hospital of Guangzhou Medical University, Guangzhou, P.R. China; 6 Department of Geriatric Medicine, Qilu Hospital of Shandong University, Jinan, P.R. China; 7 Department of Pulmonary and Critical Care Medicine, Beijing Anzhen Hospital, Capital Medical University, Beijing, P.R. China; 8 Department of Pulmonary and Critical Care Medicine, East Hospital, Tongji University School of Medicine, Shanghai, China; 9 Department of Pulmonary and Critical Care Medicine, The First Affiliated Hospital of Chongqing Medical University, Chongqing, P.R. China; 10 Department of Respiratory Medicine, Shandong Provincial Hospital Affiliated to Shandong University, Jinan, P.R. China; 11 Department of Pulmonary and Critical Care Medicine, Affiliated Hospital of Jianghan University, Wuhan, P.R. China; 12 Department of Pulmonary and Critical Care Medicine, The Affiliated Hospital of Qingdao University, Qingdao, Shandong Province P.R. China; 13 Department of Respiratory Medicine, The First People’s Hospital of Yunnan Province, Kunming, P.R. China; 14 Division of Pulmonary Vascular Disease, General Hospital of Northern Theater of Command, Shenyang, P.R. China; 15 Center for Pulmonary Vascular Diseases, State Key Laboratory of Cardiovascular Disease, Fuwai Hospital, National Center for Cardiovascular Diseases, Chinese Academy of Medical Sciences and Peking Union Medical College, Beijing, P.R. China; 16 Department of Pulmonary and Critical Care Medicine, School of Medicine, Sichuan Provincial People’s Hospital, University of Electronic Science and Technology of China, Chengdu, P.R. China; 17 Department of Respiratory Diseases, Sir Run Run ShawHospital, Zhejiang University School of Medicine, Hangzhou, P.R. China; 18 Department of Cardiology, Chinese People’s Liberation Army General Hospital, Beijing, P.R. China; 19 Departments of Pulmonary Circulation, Shanghai Pulmonary Hospital, Tongji University School of Medicine, Shanghai, P.R. China; 20 Department of Cardiology, Qilu Hospital of Shandong University, Jinan, P.R. China; 21 Department of Epidemiology and Biostatistics, School of Public Health, Peking University, Beijing, P.R. China; 22 National Center for Respiratory Medicine, State Key Laboratory of Respiratory Health and Multimorbidity, National Clinical Research Center for Respiratory Diseases, Institute of Respiratory Medicine, Chinese Academy of Medical Sciences, Data and Project Management Unit, Institute of Clinical Medical Sciences, China-Japan Friendship Hospital, Beijing, P.R. China; 23 Chinese Academy of Medical Sciences, Peking Union Medical College, Beijing, P.R. China

**Keywords:** Chronic thromboembolic pulmonary hypertension, Registry, Real-world, Multimodality treatment, Prognosis

## Abstract

**Background:**

Chronic thromboembolic pulmonary hypertension (CTEPH) is a progressive pulmonary vascular disorder with substantial morbidity and mortality, also a disease underdiagnosed and undertreated. It is potentially curable by pulmonary endarterectomy (PEA) in patients with surgically accessible thrombi. Balloon pulmonary angioplasty (BPA) and targeted medical therapy are options for patients with distal lesions or persistent/recurrent pulmonary hypertension after PEA. There is an urgent need to increase the awareness of CTEPH. Qualified CTEPH centers are still quite limited. Baseline characteristics, management pattern and clinical outcome of CTEPH in China needs to be reported.

**Methods and design:**

The CHinese reAl-world study to iNvestigate the manaGEment pattern and outcomes of chronic thromboembolic pulmonary hypertension (CHANGE) study is designed to provide the multimodality treatment pattern and clinical outcomes of CTEPH in China. Consecutive patients who are ≥ 14 year-old and diagnosed with CTEPH are enrolled. The diagnosis of CTEPH is confirmed in right heart catheterization and imaging examinations. The multimodality therapeutic strategy, which consists of PEA, BPA and targeted medical therapy, is made by a multidisciplinary team. The blood sample and tissue from PEA are stored in the central biobank for further research. The patients receive regular follow-up every 3 or 6 months for at least 3 years. The primary outcomes include all-cause mortality and changes in functional and hemodynamic parameters from baseline. The secondary outcomes include the proportion of patients experiencing lung transplantation, the proportion of patients experiencing heart and lung transplantation, and changes in health-related quality of life. Up to 31 December 2023, the study has enrolled 1500 eligible patients from 18 expert centers.

**Conclusions:**

As a real-world study, the CHANGE study is expected to increase our understanding of CTEPH, and to fill the gap between guidelines and the clinical practice in the diagnosis, assessment and treatment of patients with CTEPH.

**Registration Number in ClinicalTrials.gov:**

NCT05311072.

## Introduction

Chronic thromboembolic pulmonary hypertension (CTEPH) which classified as group 4 pulmonary hypertension (PH) in the European Society of Cardiology (ESC)/European Respiratory Society (ERS) 2022 guidelines, is a disease caused by the persistent stenosis or obliteration of pulmonary arteries by organized intraluminal thrombi and consequent pulmonary vessel remodeling [1]. The exact incidence of CTEPH after acute PE remains unknown, however, some studies reported it ranges between 0.4% and 6.2% [2]. In the USA and Europe, the crude annual incidence of crude annual full (i.e. diagnosed and undiagnosed) incidence of CTEPH were 3–5 cases per 100,000 population, while in Japan the rate was lower at 1.9 per 100,000 population [3].

Till today, CTEPH is still a disease underdiagnosed and undertreated. Only 7-29% of CTEPH cases in Europe and the United States were diagnosed in 2013, and the majority of patients were in the late stage of disease at diagnosis, with New York Heart Association functional class III/IV [3]. Therefore, there is an urgent need to increase the awareness of CTEPH. In addition, the risk assessment strategy for patients with CTEPH, especially for the patients with inoperable disease, need to be identified in registry-based studies [4].

Great progress has been made these years in the therapeutic strategy of CTEPH. CTEPH is potentially curable by pulmonary endarterectomy (PEA) in patients with surgically accessible thrombi in the main, lobar or segmental pulmonary arteries [5]. However, PEA is not feasible in all patients; up to a half of the patients are considered inoperable and up to 51% develop persistent/recurrent PH after undergoing PEA [6–9]. Balloon pulmonary angioplasty (BPA) is an option for CTEPH patients with lesions in the distal pulmonary arteries. The specific medication for pulmonary arterial hypertension (PAH) can also be used in patients with CTEPH, since they have similar pulmonary vessel remodeling. Due to the progress in the surgical and interventional therapy technic as well as the development of PAH specific therapy, the prognosis and life quality of patients with CTEPH have been improved. There is still a gap between the normalized management and real-world clinical practice. Moreover, qualified CTEPH centers, in which PEA and BPA can be performed with satisfactory quantity and quality, are quite limited in China. The existing studies on CTEPH have the limitations with long time-span and relative small sample size, in which limited patients received BPA or novel target therapy. Thus, they could not accurately reflect the current status in the new era of multimodality therapy in CTEPH.

In a large international CTEPH registry with 679 patients, the 3-year survival was 89% in operated patients and 70% in non-operated patients, respectively [10]. The 5-year and 10-year survival was 82 and 75%, respectively, reported by the University of California, San Diego in the United States, known as the most experienced center in PEA [11]. The therapeutic effect and long-term outcome of patients with CTEPH need to be identified in a large cohort in Asia. High-quality studies in the real world are required to further increase the understanding of CTEPH, including the clinical characteristics, management pattern, outcomes of CTEPH and related risk factors.

## Methods

### Study objective and design

**Fig. 1:**
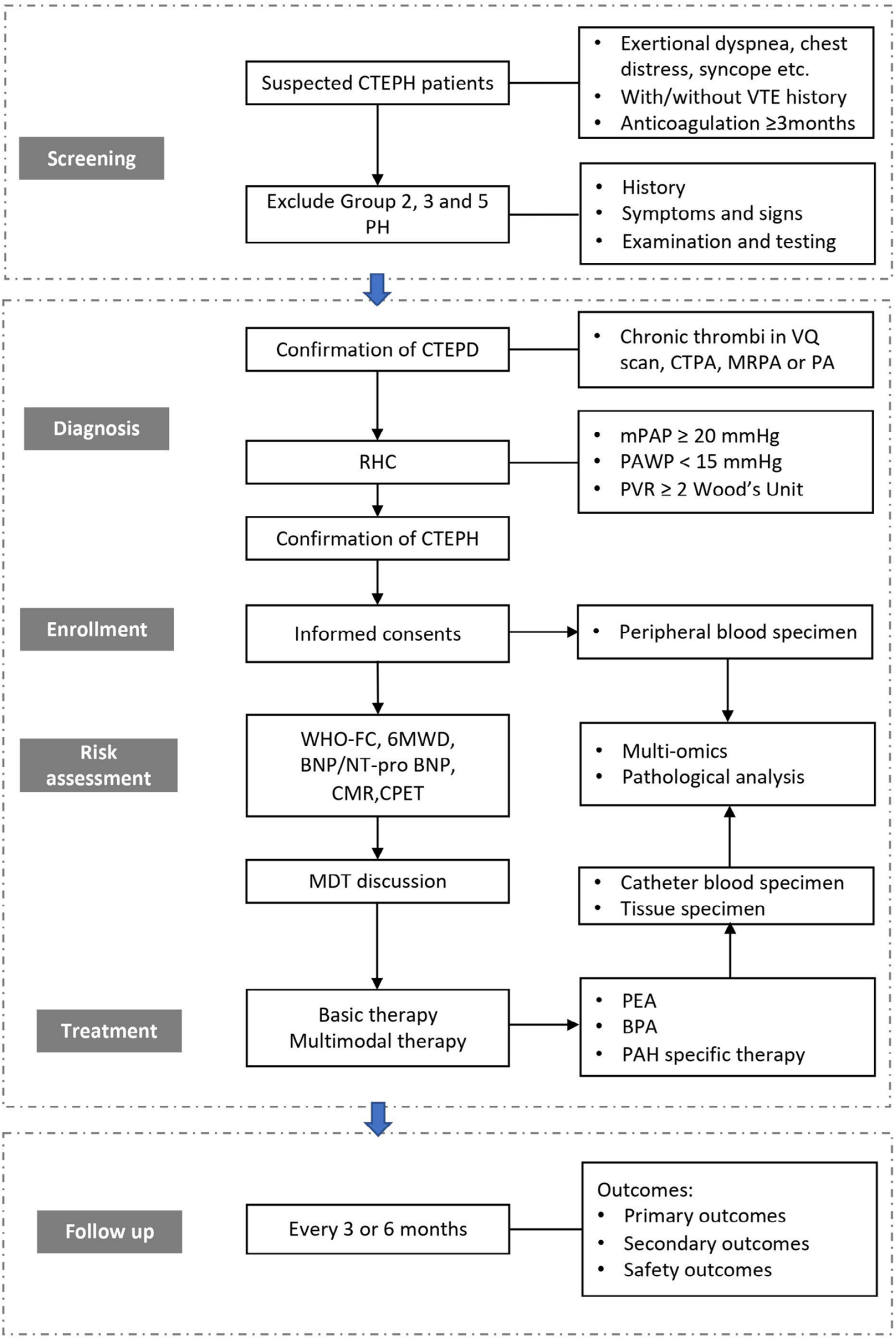
Design and flowchart of the study CTEPH: chronic thromboembolic pulmonary hypertension; VTE: venous thromboembolism; CTEPD: chronic thromboembolic pulmonary disease; VQ: ventilation/perfusion; CTPA: computer tomography pulmonary angiography; MRPA: magnetic resonance pulmonary angiography; PA: pulmonary angiograph; RHC, right heart catheterization; mPAP: mean pulmonary arterial pressure; PAWP: pulmonary artery wedge pressure; PVR: pulmonary vascular resistance; WHO-FC: WHO functional class; 6MWD: 6-minute walking distance; CMR: cardiac magnetic resonance; CPET: cardiopulmonary exercise testing; MDT: multidisciplinary team; PEA: pulmonary endarterectomy; BPA: balloon pulmonary angioplasty; PAH: pulmonary arterial hypertension

The CHinese reAl-world study to iNvestigate the manaGEment pattern and outcomes of chronic thromboembolic pulmonary hypertension (CHANGE) study aims to investigate the demographic and clinical characteristics, the management pattern, the outcomes of CTEPH and related risk factors. It’s a multicenter, prospective observational study, which enrolls and follows up newly-diagnosed CTEPH patients in a longitudinal setting. Their baseline information, therapeutic pattern and prognosis were collected. Design and flowchart of the study are presented in Fig. 1. The patients enrolled are followed up for at least 3 years with every 3 or 6 months.

### Study hospitals and patient enrollment

In the CHANGE-CTEPH study, there are 18 centers settings from 9 provincial administrative regions in mainland China. All of tertiary and teaching hospitals with rich experience in the management of abundant patients with PH. Most of the participating centres were referral centres for PEA and/or BPA.

Consecutive patients who are ≥ 14 year-old, diagnosed with CTEPH and willing to sign the informed consent are enrolled. The diagnosis of CTEPH needs to meet the following criteria: (1) mismatched perfusion defects on ventilation/perfusion (VQ) scintigraphy and specific diagnostic signs for CTEPH seen by conventional pulmonary angiography (PA), computer tomography pulmonary angiography (CTPA), or magnetic resonance pulmonary angiography (MRPA) such as ring-like stenosis, webs/slits and chronic total occlusions (pouch lesions or tapered lesions), with effective anticoagulation for at least 3 months; (2) precapillary PH proved by right heart catheterization (RHC), defined as mean pulmonary artery pressure (mPAP) ≥ 20mmHg with pulmonary artery wedge pressure (PAWP) ≤ 15 mmHg and pulmonary vascular resistance (PVR) > 2 Wood’s Units.

The exclusion criteria: (1) diagnosed with other diseases in group 4 PH, like systemic vasculitis without connective tissue disease and pulmonary tumors; (2) informed consent are not available; (3) not able to complete the follow-up.

### Baseline assessment

All the patients enrolled receive imaging examinations (including VQ scan, CTPA, MRPA or PA) and RHC at baseline, in order to confirm the diagnosis of CTEPH. The baseline information is collected, including the demographics, symptoms and signs, past medical history, laboratory findings (complete blood cell count, coagulation test, liver and renal function, brain natriuretic peptide (BNP)/N terminal-proBNP (NT-proBNP), thrombophilia test et al.), electrocardiogram, ultrasonic cardiogram, imaging examinations and RHC data. The WHO-functional classification (WHO-FC), 6-minute walking distance (6MWD) and initial therapy (including medication, surgery and interventional therapy) are also recorded.

### Biospecimen and biobank

Blood samples of enrolled eligible patients who are able to provide written consent are collected via local well-trained investigators according to critical standard operating procedure. The biospecimen will be centrifuged, divided into aliquots (plasma, serum, or blood cells, respectively) as each participating center and transported with dry ice to the central biobank, which was established within China-Japan Friendship Hospital, and then stored at − 80 °C for further genetic and molecular analyses.

The tissue from PEA of enrolled eligible patients who received PEA is also stored in the central biobank in China-Japan Friendship Hospital after the histopathological observation. Human pulmonary arterial endothelial cells and smooth muscle cells are isolated, cultured and cryopreserved for further research.

### Therapeutic strategy

All the patients received a life-long anticoagulation, with warfarin, direct oral anticoagulants （DOACs） or low-molecular-weight heparin. The therapeutic strategy is made by a multidisciplinary team (MDT) consist of PH specialist, PEA surgeon, interventionist, radiologist experienced in thoracic imaging, anesthesiologist and intensive care specialist. The patients with surgically accessible thrombi in the main, lobar or segmental pulmonary arteries and who are willing to receive surgery will receive PEA. The patients who are considered inoperable in the first center will go for consultation in another PH center. BPA and PAH target medication are used in patients who are inoperable or unwilling to receive surgery, and who have recurrent PH after PEA, based on the experience of pulmonologist, cardiologist, and interventional radiologist in each center. Diuretics and cardiotonics are selectively used in the patients with right heart dysfunction. Patients whose cardiopulmonary function is stable also receive rehabilitation during hospitalization and at home.

### Follow-up and endpoints

The patients receive regular follow-up every 3 or 6 months after enrollment for at least three years. For each follow-up, they receive inquiry, physical examination, 6-minute walking test (6MWT), blood tests, electrocardiogram, ultrasonic cardiogram, VQ scan or CTPA and RHC. Cardiopulmonary exercise testing (CPET) and cardiac MR are arranged in selected patients. The therapeutic strategy is modified according to the assessment of each follow-up. If the patients are admitted to the hospital unexpectedly, the cause of hospitalization, the change of symptoms and treatment since last visit, 6MWD, WHO-FC and the change of PVR will be recorded.

The primary outcomes include all-cause mortality and changes in functional and hemodynamic parameters from baseline. The secondary outcomes include the proportion of patients experiencing lung transplantation, the proportion of patients experiencing heart and lung transplantation, and changes in health-related quality of life. The study outcomes were listed in Table 1.

**Table 1:** Primary, secondary, and safety outcomes

Outcomes	Main indicators
**Primary outcomes**	
all-cause mortality	
the change of functional parameters from baseline	functional parameters include 6MWD, WHO-FC and BNP/NT-proBNP
the change of hemodynamic parameters from baseline	hemodynamic parameters include mPAP, PVR, CO and CI
**Secondary outcomes**	
lung or heart and lung transplantation	proportion of patients experiencing lung or heart and lung transplantation due to severe right heart failure during the study
changes of health related quality of life	change of quality of life measured with the EQ-5D-5L or EmPHasis-10
**Safety outcomes**	
bleeding	fatal bleeding, major bleeding, clinically relevant non-major bleeding, based on the ISTH definition [15, 16]
symptomatic recurrence of DVT or PE	reappearance of clinical manifestations or symptoms associated with DVT or PE after a previous episode or treatment, include leg pain, swelling, warmth, redness, or shortness of breath, etc.
complication of treatment interventions (PEA or BPA)	complications include lung injury, haemoptysis, residual pulmonary hypertension, etc.

6MWD: 6-minute walk distance; WHO-FC: WHO-functional class; BNP: brain natriuretic peptide; NT-proBNP: N terminal-probrain natriuretic peptide; mPAP: mean pulmonary arterial pressure; PVR: pulmonary vascular resistance; CO: cardiac output; CI: cardiac index; EQ-5D-5L: 5-level EQ-5D version; ISTH: international society on thrombosis and haemostasis; DVT: deep venous thrombosis; PE: pulmonary embolism; PEA: pulmonary endarterectomy; BPA: balloon pulmonary angioplasty

### Quality control, data management and monitoring

We designed an electronic data capture (EDC) system and an electronic case report form (eCRF) to capture and check all data. Patient information is collected and then entered into the CHANGE platform which was built on a secure Website (https://edc.einmatrix.com/studies). The investigators who enter information into the EDC system are responsible for ensuring accuracy and completeness of information. All electronic data are protected by encryption. Data management and monitoring are carried out by a dedicated project and data management team according to predefined procedures to avoid bias. Once abnormal or missing values are detected, queries will be sent by the EDC system automatically to local investigators to check and revise the data. To guarantee the data quality and follow-up rate, trained clinical research coordinators from the project and data management team will visit the participating centers if they have a large proportion of missing value or a lower follow-up rate to check the consistency between registered information and original medical records in randomly selected patients.

### Statistical analysis plan

Categorical variables will be presented as numbers and percentages. Normally distributed continuous variables will be shown as means ± standard deviations(SD), while non-normally distributed continuous variables will be presented as medians together with 25% and 75% percentiles. Multivariable logistic regression models will be used to determine independent factors and their magnitudes of associations with clinical outcomes during hospitalization. Time-to-event outcomes will be presented using Kaplan-Meier methods and hazard ratios and corresponding 95% confidence intervals (CI) will be derived from the Cox proportional hazards regression models. In addition, enrolled patients will be stratified into subgroups according to different study stages, age of patients, or therapeutic options etc. for further analyses. Statistical significance will be established at 2-tailed *P* value < 0.05. All statistical analyses will be performed in SAS 9.4 (SAS Institute Inc. Cary, NC, USA).

### Ethics and informed consent

The study protocol has been approved by the China-Japan Friendship Hospital ethics committee (2022-KY-085). All the collaborating centers received approvals from their local ethics committee. All patients enrolled have provided written informed consent to their participation, as required by the ethics committee according to the Declaration of Helsinki and local regulatory policies at each center. The CHANGE study has been registered in the ClinicalTrials.gov with an identifier of NCT05311072.

## Discussion

The CHANGE study is designed to provide data on the management pattern and outcomes of CTEPH in China, as well as to reveal the intrinsic etiology and pathogenesis of the disease. It is a national prospective multicenter ongoing registry on the diagnosis, initial treatment, long-time management and prognosis of CTEPH. It was launched since 2016 in China, and until 31 December 2023 it has enrolled 1500 potentially eligible patients.

Up-to-date, the study has the largest sample size set in the real-world in China and continues to consecutively recruit eligible patients, which offers efficient statistical power in data analyses and robust conclusion interpretation. As of December 2024, the enrolled CTEPH patients in the CHANGE registry have received regular follow-ups every three or six months for at least 3 years, which provides opportunities to investigate the long-term sequelae of CTEPH.

Since two decades ago, registry studies on CTEPH have been conducted all over the world, predominantly distributed in Europe and the United States. There are also CTEPH registries in Asian countries including China. Several representative ongoing large registries on CTEPH are summarized in Table 2. The majority of these registries launched before 2010, when the surgical and interventional therapeutic technique was still immature and the application of PAH-specific drugs were quite limited.

**Table 2:** Preexisting registry studies on CTEPH

	Inclusion criteria	Number of CTEPH patients	Number of PEA patients	Enrolling time
International CTEPH Association (ICA) [12]	newly diagnosed patients fulfilling the following criteria: (1) be willing to provide informed consent, (2) have been treated with anticoagulation for at least 3 months before diagnosis of CTEPH established at the RHC with mPAP ⩾25mmHg at rest, abnormal ventilation/perfusion scan, pulmonary angiogram, CTPA or MRPA confirming chronic thromboembolic obstructions	1010	649	February 2015- September 2016
United States [17]	patients newly diagnosed (< 6 months) with CTEPH (mPAP ≥ 25mmHg in RHC and radiologic confirmation of CTEPH)	750	566	2015–2018
Europe and Canada [18]	newly diagnosed (≤ 6 months) consecutive patients with CTEPH (mPAP ≥ 25mmHg in RHC at rest or ≥ 30 mmHg after exercise and a PAWP ≤ 15 mmHg) who did not receive PAH-targeted treatment before diagnosis	679	386	February 2007- January 2009
Europe (COMPERA) [14]	(1) treatment-naive patients newly diagnosed with CTEPH or residual pulmonary hypertension after PEA, with data from baseline and at least one follow-up visit available; (2) mPAP ≥ 25 mmHg and PAWP ≤ 15 mmHg at the time of diagnosis; (3) no PEA or BPA during follow-up; and (4) at least three of the six listed variables available at baseline	561	0	January 2009- December 2017
China [19]	consecutive incident and prevalent patients diagnosed with CTEPH: (1) after at least 3 months of effective anticoagulation; (2) mPAP ≥ 25 mm Hg and PAWP ≤ 15 mm Hg at rest in RHC; (3) mismatch on ventilation/perfusion scintigraphy with at least one large perfusion defect in one segment or in two subsegments; or evidence of pulmonary vascular lesions on CTPA and/or MRI or pulmonary angiography.	593	81	August 2009- July 2018
Germany [20]	adult patients ≥ 18 years of age with a newly established CTEPH diagnosis	392	197	2016
Swiss [21]	patients newly diagnosed with PH (mainly PAH/ CTEPH) confirmed in RHC	383	90	January 2001- June 2019
Japan [22]	patients with CTEPH who underwent BPA	308	0	November 2004- March 2013
South Korea [23]	(1) after at least 3 months of effective anticoagulation; (2) mPAP > 25 mmHg with PAWP ≤ 15 mmHg, measured by RHC, or a right ventricular systolic pressure > 40 mmHg estimated by echocardiography; (3) at least one segmental perfusion defect detected by either lung scanning, multi-detector CTPA, or pulmonary angiography	134	28	September 2008- October 2011
Russia [24]	patients aged > 18 years with newly diagnosed CTEPH	206	66	January 2012- January 2019
Saudi Arabia [25]	mPAP ≥ 25mmHg and PAWP ≤ 15mmHg in the presence of occlusive thrombi in the elastic pulmonary arteries, mismatched perfusion defects on lung scan and specific diagnostic signs for CTEPH seen by multidetector CTPA, MRI or pulmonary angiography, after ≥ 3 months of anticoagulation	64	43	October 2007- March 2018

CTEPH, chronic thromboembolic pulmonary hypertension; RHC, right heart catheterization; mPAP, mean pulmonary arterial pressure; CTPA, computer tomography pulmonary angiography; MRPA, magnetic resonance pulmonary angiography; PAWP, pulmonary artery wedge pressure; PEA, pulmonary endarterectomy; BPA, balloon pulmonary angioplasty; MRI, magnetic resonance imaging; PAH, pulmonary arterial hypertension

It’s notable that there were fewer patients who received PEA in CTEPH registries in Asian countries, that implies surgical therapy need to be further extended and disseminated. The registry data of international CTEPH association (ICA) [12] highlight regional differences among different countries/areas, regarding patient characteristics and therapeutic approaches, diagnostic imaging and treatment allocations between operable and inoperable patients. The arena for optimal management of CTEPH has changed dramatically over the last decade accompanied with the progress of BPA technique and PAH-specific therapies. Registries on CTEPH in the new era are needed to reveal the impact of the progress made in therapeutic strategy on the prognosis of patients with CTEPH.

The centers in this registry are expert CTEPH centers in China, which comprehensively integrate multi-disciplinary teams for multimodal interventions, including PEA, BPA and PAH-specific agents. Data on the baseline characteristics, management pattern and outcomes of patients with CTEPH from these centers distributed in different provinces of China have good representativeness and creditability.

There is currently no recommendation regarding risk stratification in patients with inoperable CTEPH. However, as a common and well-validated method used in patients with PAH, the REVEAL risk score has shown utility as a prognostic tool in CTEPH [13]. In a recent study, the Swedish/COMPERA method also shows the potential to predict mortality in patients with inoperable CTEPH [14]. We will assess these risk stratification models in patients with inoperable CTEPH, and also explore and verify a new risk stratification strategy to guide the management of these patients.

The multimodality treatment has been applied more widely in patients with CTEPH in recent years. Numerous unanswered questions and challenges continue to arise in the new era of multimodal CTEPH treatment. There are several scenarios of the multimodality therapy for CTEPH: (1) some patients with inoperable CTEPH receive sequential or simultaneous treatment with targeted medical therapy and BPA; (2) BPA and/or targeted medical therapy have been applied in patients with persistent or recurrent PH after PEA; (3) patients with operable CTEPH but severe hemodynamic impairment could receive PEA after several sessions of BPA ± targeted medical therapy with reduced surgical risk; (4) combination of PEA and BPA might be performed in selected patients with high-risk hemodynamics with unilateral operable and contralateral inoperable disease. It remains unclear which is the best sequence and interval of the hybrid of therapeutic strategy for selected patients. We will try to find the answer to these questions in the real-world study.

In the CHANGE registry, we will also focus on the medical treatment in patients with CTEPH in recent years, especially PAH-specific drugs and anticoagulants. Up to now, riociguat was the only agent approved for the treatment of CTEPH and used as first choice for all CTEPH patients who need PAH-specific therapy. However, riociguat was not available or not included in health insurance in some areas, that limit its application in clinical practice. In addition, other PAH-specific drugs, such as macitentan and subcutaneous treprostinil, also exhibited promising efficacy in CTEPH. PAH-specific drugs which appeared on the market earlier, like sildenafil, tadalafil, bosentan and ambrisentan, were chosen to treat patients with CTEPH when riociguat was not available or well tolerated. It remains to be illustrated which kind of PAH-specific agent and whether combined PAH-specific therapy bring benefit for patients with CTEPH. All the guidelines on CTEPH recommend life-long anticoagulation for CTEPH patients without contradiction. Nonetheless, the effect and safety of DOACs in CTEPH is still controversial and need to be illustrated in both randomized controlled trials and real-world studies.

The establishment of biospecimen and biobank is a new bright spot in this registry. The blood sample will be used in the multi-omics research, including the genomics, transcriptomics and proteomics of CTEPH. The sample from PEA of CTEPH patients will be used in the clinical pathological analysis and single-cell sequencing. The primary human pulmonary arterial endothelial cells and smooth muscle cells will be isolated and used in the basic research to further illustrate the pathogenesis of CTEPH. This part is a critical approach to link the phenotype to the endotype of patients with CTEPH.

## Summary

Despite advances in the treatment of CTEPH, there are still gaps remaining to be filled in our understanding. The CHANGE registry is an ongoing multicenter study to investigate the management pattern and outcomes of patients with CTEPH in the new era of multimodal CTEPH treatment. The demographic and clinical characteristics, management pattern, clinical outcomes of CTEPH and related risk factors will be analyzed in this real-world study. We aim to improve the clinical practice in the diagnosis, assessment and treatment of patients with CTEPH via the conduction of clinical study and the construction of research system.

## Data Availability

Not applicable.
